# Concept of Normativity in Multi-Omics Analysis of Axon Regeneration

**DOI:** 10.3390/biom14070735

**Published:** 2024-06-21

**Authors:** Isabella Moceri, Sean Meehan, Emily Gonzalez, Kevin K. Park, Abigail Hackam, Richard K. Lee, Sanjoy Bhattacharya

**Affiliations:** 1 Miami Integrative Metabolomics Research Center, Bascom Palmer Eye Institute, University of Miami, Miami, FL 33136, USA; igm243@miami.edu (I.M.); sdm109@miami.edu (S.M.); emilygonz07@gmail.com (E.G.); ahackam@med.miami.edu (A.H.); rlee@med.miami.edu (R.K.L.); 2 Graduate Program in Molecular Cellular Pharmacology, University of Miami, Miami, FL 33136, USA; 3 Department of Ophthalmology, University of Texas Southwestern Medical Center, 5323 Harry Hines Blvd., Dallas, TX 75390, USA; kyung.park@utsouthwestern.edu

**Keywords:** axon regeneration, multi-omics, normalization, glaucoma

## Abstract

Transcriptomes and proteomes can be normalized with a handful of RNAs or proteins (or their peptides), such as GAPDH, β-actin, RPBMS, and/or GAP43. Even with hundreds of standards, normalization cannot be achieved across different molecular mass ranges for small molecules, such as lipids and metabolites, due to the non-linearity of mass by charge ratio for even the smallest part of the spectrum. We define the amount (or range of amounts) of metabolites and/or lipids per a defined amount of a protein, consistently identified in all samples of a multiple-model organism comparison, as the normative level of that metabolite or lipid. The defined protein amount (or range) is a normalized value for one cohort of complete samples for which intrasample relative protein quantification is available. For example, the amount of citrate (a metabolite) per µg of aconitate hydratase (normalized protein amount) identified in the proteome is the normative level of citrate with aconitase. We define normativity as the amount of metabolites (or amount range) detected when compared to normalized protein levels. We use axon regeneration as an example to illustrate the need for advanced approaches to the normalization of proteins. Comparison across different pharmacologically induced axon regeneration mouse models entails the comparison of axon regeneration, studied at different time points in several models designed using different agents. For the normalization of the proteins across different pharmacologically induced models, we perform peptide doping (fixed amounts of known peptides) in each sample to normalize the proteome across the samples. We develop Regen V peptides, divided into Regen III (SEB, LLO, CFP) and II (HH4B, A1315), for pre- and post-extraction comparisons, performed with the addition of defined, digested peptides (bovine serum albumin tryptic digest) for protein abundance normalization beyond commercial labeled relative quantification (for example, 18-plex tandem mass tags). We also illustrate the concept of normativity by using this normalization technique on regenerative metabolome/lipidome profiles. As normalized protein amounts are different in different biological states (control versus axon regeneration), normative metabolite or lipid amounts are expected to be different for specific biological states. These concepts and standardization approaches are important for the integration of different datasets across different models of axon regeneration.

## 1. Introduction

Neurons are the most asymmetric cells in human bodies. Axons are very long appendages that connect one neuron with the other for electrical signal transduction and they are major contributors to the asymmetry of neurons. Irreversible and progressive loss of peripheral vision occurs due to damage to and the loss of axons from retinal ganglion cells (RGCs) [1,2] within the optic nerve. The axons of peripheral RGCs must traverse the longest distances; therefore, they are the most vulnerable and are usually lost first, as reflected by the initial peripheral vision loss due to static perimetry in glaucoma. Studies of human cadaveric eyes as well as animals models suggest that RGCs do not remodel immediately but survive in the retina for a long time [3,4]. The regeneration of axons holds the promise to reconnect RGC axons with brain neurons and restore vision. The best-studied paradigm is optic nerve crush (ONC) and then pharmacological or biophysical stimulation, promoting the regeneration of axons from existing RGCs. We utilize a channel rhodopsin transgenic mouse for axon regeneration with biophysical rhythmic light stimulation after ONC [5,6], with regeneration promoted for different lengths of time. We present axon regeneration as a model system in order to present some advanced methods of protein normalization. We also present concepts of how to quantitatively compare metabolites and lipids (small molecules) across the whole repertoire of these molecules. 

Transcriptomes remain the best-studied omes for axon regeneration thus far. This is due to the robust nucleic acid’s chemistry and the widespread availability of microarray platforms, which have experienced significant advancements over the past few decades. Nucleic acid chemistry also presents the advantages of amplification, which is not available for the analysis of other omes. However, transcriptomic analysis comes with stability and translational concerns. The half-life of mRNAs varies among organisms and can be as short as 3 min in *E. coli* [7]. Additionally, increased mRNA stability does not translate to increased protein abundances. Although transcriptomic profiles are more accessible and available at higher numbers, multi-omic normalization based on transcriptome values poses its own challenges. In yeast, careful studies have shown that the correlation of protein and mRNA is at best 38% [8]. Proteins are more closely related to biological functions and normalization based on a proteome is likely to be reflective of its functions. 

Mass spectrometry-based proteomics, lipidomics, and metabolomics remain the most versatile and robust platforms for the analyses of these omes. Metabolomics is the more time-scale-variant (fleeting) ome, but may present great opportunities for promoting axon regeneration. The pharmacologic experience of multiple ocular drug development fields (such as for age-related macular degeneration) and other neurodegeneration fields (for example, demyelinating diseases) suggests that multiple-organism comparisons are more helpful for identifying targets as well as molecules suitable for treatment across various patient populations. Most diseases have age-related, genetic, environmental, and other variables. No model organism perfectly represents all components, as found in human patient populations. The biochemical individuality of human patients is also a factor in determining responsiveness to the molecules identified for treatment. Thus, multi-organism (multiple separate pharmacologic treatment) and multi-omic comparisons are necessary. The need to assess different time points where different degrees of axon regeneration occur is also important. Due to the higher overall stability of some housekeeping proteins and their better reflection of functionality, a normalized proteome (based on a few proteins) is advantageous, but more advanced approaches than those currently available are needed. The small molecules, metabolome, and lipidome suffer from non-linearity, even in the shortest range of the mass spectrum. Nuclear magnetic resonance (NMR) currently identifies and quantifies only a small repertoire of these molecules. For broader profiling, mass spectrometry offers a more practical approach; however, due to the large number of molecules spread over the dynamic range of the first mass analyzer in any tandem mass spectrometer, normalization is not possible, even with large numbers of standards across the range of molecules. We also present the concept of the proteome-normalized range of metabolites and/or lipids associated with ONC and different stages of axon regeneration as being representative of two biological states as an example of the applicability of this concept (normativity) across other biological spectrums.

## 2. Materials and Methods

The data presented were generated from mass spectrometry-based analysis studying the effects of optogenetic stimulation post optic nerve crush injury in Thy1-ChR2-eYFP (regeneration) and C57BL/6J (control) mice. Optogenetic models prove that the manipulation of RGC growth through the expression and stimulation of channel rhodopsin using the RGC-specific Thy-1 promoter demonstrates axon regeneration. With batch variability and false positives remaining a consistent problem when performing quantitative proteomic experiments, we introduce the concept of proteomic normalization and its relationship to the normativity of all other omes.

### 2.1. Proteins

#### 2.1.1. Experimental Design

Overall, 6 experimental conditions were studied with 6 biological replicates each (n = 6) for a total of 36 optic nerves collected from Thy1-Chr2-eYFP and C57BL/6J mice. The samples were split evenly into three batches of 12 plex TMT for relative quantification. The protein from the optic nerve tissues was extract, digested, and isobarically labeled with TMT (Thermo Scientific™, Waltham, MA, USA). Each plex was pooled together for untargeted liquid chromatography–mass spectrometry analysis.

#### 2.1.2. Protein Extraction

Experimental mice and wild-type control mice were euthanized, and optic nerves were collected via dissection. The optic nerve samples were minced in a protein extraction buffer containing 10 mM TEAB pH 8.5, 50 mM NaCl and 0.1% SDS and three synthetic non-mammalian peptide standards (Regen III) for extraction efficiency measurement. The Regen III peptides are LLO: GYKDGNEYI, SEB: KKKVTAQELD, and CFP: EISTNIRQAGVQYSR. Each standard was spiked into each sample for a total concentration of 36 μM per standard. The samples were vortexed and centrifuged, and supernatants were placed into a new tube. After extraction had been carried out, protein amounts were estimated, using dot blot densitometry and ImageJ to normalize to 70 µg/µL across all samples. The protein was denatured, reduced, and alkylated by 2% SDS, 110 mM TCEP, and 84 mM iodoacetamide, respectively. Samples were trypsin-digested overnight. Prior to labelling, three samples of mass spec-grade pre-digested bovine serum albumin (BSA) were prepared at a concentration of 150 pmol. These samples were included to account for differences in labelling efficiency between TMT batches. All samples, including the three BSA standards, were labelled using 3 sets of 13 tags from a 16 plex TMT (Tandem Mass Tag) kit for quantification. After the combination and drying of all peptide samples, each combined TMT sample (including one BSA-labelled standard) was spiked with two additional human peptide standards containing isobaric labels (Regen II). The Regen II peptides were as follows: A1315: DRV(U-13C5 15N)YI(U-13C615N)HPFHL and HH4B: SGRGKGGKGLGKGGAKRHRKVLRGGK(Biotin). The isobaric carbons were 13C and nitrogen was 15N as indicated. Regen V peptides: Regen III and Regen II (>98% purity) were synthesized using the synthetic peptide services of Anaspec, Fremont, CA, USA. The final concentration of Regen II was 54 µM. These standards were spiked in directly before mass spectrometry analysis for used as ionization controls.

#### 2.1.3. Untargeted Liquid Chromatography and Mass Spectrometry

Dried samples were reconstituted in 48 μL of 2% acetonitrile in water with 0.1% formic acid and then sonicated in an ultrasonic water bath for 15 min for total solubilization. Samples were transferred to their respective autosampler vials. Proteins were then separated using a Thermo Scientific™ Easy-nLC 1000 system and 1 μL of sample was injected into an Easy-Spray HPLC column (Thermo Scientific™, Waltham, MA, USA, ES900). The flow rate was 300 nL/min. Mobile phase A consisted of water with 0.1% formic acid (*v*/*v*) and mobile phase B was acetonitrile with 0.1% formic acid (*v*/*v*). The column’s temperature was 55 °C.

Samples were ionized and detected using an EASY-Spray™ source coupled to a Q Exactive™ mass spectrometer (Thermo Scientific™, Waltham, MA, USA). The spray voltage was set to 1.9 kV and the capillary temperature was set to 300 °C. The S-Lens RF level was set to 60.0. For full scans, the mass range was 375–1400 *m*/*z*, the resolution was 70,000, and there was 1 microscan. The AGC target was 3 × 10^6^ and maximum injection time was 50 ms. For dd-MS2, the mass range was 200–2000 *m*/*z*, the resolution was 35,000, the AGC target was 1 × 10^5^, and the maximum injection time was 100 ms. The instrument was set to Top 10, the isolation window was set to 1.2 *m*/*z*, and the NCE was set to 32. The intensity threshold was 2.04 × 10^4^ and dynamic exclusion was 30.0 s.

#### 2.1.4. Protein Identification

The raw scans were processed via Proteome Discoverer 3.0.1 using a SEQUEST HT engine. The data were searched against Mus musculus entries (Swiss-Prot + TrEMBL, UniProt 1/15/2024). The max missed cleavage site was set to 2 and the minimum peptide length was set to 6. Precursor mass yolerance was set to 10 ppm and fragment mass tolerance to 0.02 Da. Post-translational modifications for experimental proteins included oxidation, acetylation, carbamidomethylation, and TMTpro. The normalization was performed in relation to the total peptide amount and confidence was medium. Target FDR was applied: 0.01 (strict) and 0.05 (relaxed). A 13 plex TMT label reagent kit (modified from a 16 plex Thermo Scientific Kit) was used as the quantification method. Two additional local databases were created: one contained the regen peptide internal standard peptide sequences, and the other contained BSA peptide sequences. The identification of the Regen V and BSA standards was ran in a separate Proteome Discoverer workflow targeted at identifying the internal standards separately from experimental proteins. Post-translational modifications of the regen peptides (biotin, carbon-13 and nitrogen-15) were added during this step to ensure the correct identification of the modified standards.

#### 2.1.5. Regen Peptide Normalization

Regen V peptide abundances were normalized by biological replicate by batch to their respective values, given in µg, based on the known volume spiked in each tagged sample. Both pre-extraction peptides (Regen III) and post-extraction peptides (Regen II) were individually normalized to trypsin-digested BSA and a correction factor was calculated based on the BSA normalization. 

Regen peptide BSA normalization: (raw peptide abundance × trypsin-digested BSA concentration (µg))/(peptide concentration (µg) × BSA raw abundance).

#### 2.1.6. Protein Normalization and Pathway Analysis

The identified proteins were filtered to obtain abundance values in at least two biological replicates. A total of 1819 high- and medium-confidence proteins were identified after filtering. Plexes were separated and analyzed by batch. The raw abundance values were normalized to 100 pmol of trypsin-digested BSA. The BSA-normalized protein levels were multiplied by the correction factor for each sample in each batch. The normalized values were then taken from each batch and averaged together by sample, resulting in one value per protein per sample for all of the plexes. 

Peptide normalization: ((peptide raw abundance × trypsin-digested BSA concentration (µg))/(BSA raw abundance)) × (BSA-normalized Regen II/BSA-normalized Regen III). 

Statistical *t*-tests were run between regenerative and nonregenerative groups to find significant proteins. Only significant proteins were used for pathway analysis. Protein IDs were converted into their KEGG abbreviations and uploaded individually via KEGG Mapper for a complete list of identifiable pathways.

### 2.2. Lipids

#### 2.2.1. Experimental Design

Channel rhodopsin mice and controls were divided into four experimental conditions, each with six biological replicates and each for a total of 24 optic nerves. Optic nerves were collected, and a lipid extraction was performed with the Bligh and Dyer method. The samples were analyzed in positive and negative ion modes (2 scans each) with untargeted liquid chromatography–mass spectrometry (LC-MS/MS). 

#### 2.2.2. Lipid Extraction

A modified Bligh and Dyer method was used for lipid extraction with LC-MS-grade solvents. Dissected optic nerves were stored at −80 °C and treated with 6 mL of methanol and 3 mL of chloroform per sample. The samples were vortexed and sonicated in ultrasonic baths for 2 min and incubated overnight at 48 °C. This was followed by the addition of 2 mL of water and 1.5 mL of chloroform. For protein and lipid separation, the samples were vortexed again for 2 min and centrifuged at 3000 RCF for 15 min. The lower phase was collected and dried completely in a centrifugal vacuum concentrator.

#### 2.2.3. Untargeted Liquid Chromatography and Mass Spectrometry

Dried lipid samples were reconstituted in 60 µL of isopropanol–acetonitrile 1:1 (*v*/*v*) and sonicated for 20 min in an ultrasonic water bath for total solubilization. Samples were analyzed by using liquid chromatography electrospray–tandem mass spectrometry (LC-MS/MS). The instruments used were the Accela 6000 HPLC system and an orbitrap mass spectrometer (Q-Exactive, Thermo Scientific, Waltham, MA, USA). A Thermo Acclaim 120 C18 (150 × 2.1 mm, 3 µm) column (Thermo Scientific™, Waltham, MA, USA) was run with methanol–water 60:40 (*v*/*v*) and 10 mM ammonium acetate and methanol chloroform 60:40 (*v*/*v*) with 10 mM ammonium acetate, measured as solvent A and B, respectively. The heated electrospray ionization source (HESI) was used with the following settings: spray voltage 4.415 kV, vaporization temperature 275 °C, and auxiliary gas flow 15 arbitrary units.

#### 2.2.4. Lipid Identification and Analysis 

The raw scans were processed with LipidSearch 5.1 with the following parameters: a Precursor Tolerance of 5.0 ppm, Product Tolerance of 8.0 ppm, Product Threshold of 1.0 ppm, Noise Threshold of 3.0, Intensity Ratio Threshold of 1.5, and Valid Peak Rate Threshold of 0.5. The alignment of positive and negative lipid species was individually performed with a Retention Tolerance of 0.05 min and Retention Correction Tolerance of 0.5 min. Deuterium standards were identified and used for the normalization of each lipid class within the sample. Both positive and negative lipid identifications were aligned and consolidated for further analysis.

Metaboanalyst 6.0 was used to further analyze the data. Missing values were excluded from analysis; data were filtered using Interquartile Range (IQR) and transformed using log transformation (base 10). Volcano plots were generated for the identification of significance in different sample comparisons, using a *p* value threshold of 0.5 and a fold-change threshold of 2.0. Significant lipid species abbreviations were uploaded to LIPEA for pathway analysis and converted into KEGG identifications. To confirm the pathway identification of LIPEA, the KEGG identifiers were also uploaded to the KEGG Mapper—Search tool for further analysis.

### 2.3. Metabolites

#### 2.3.1. Experimental Design

Channel rhodopsin and wild-type mice were divided into 6 experimental conditions, with three biological replicates each. Due to the small tissue size of murine optic nerves, the nerves from three mice per condition were pooled prior to performing extraction. A total of 54 optic nerves were collected. The metabolites were identified via untargeted liquid chromatography–mass spectrometry profiling. Each sample was analyzed in positive and negative ion modes (3 scans each).

#### 2.3.2. Metabolite Extraction

Metabolite extraction was carried out in a 4 °C room and samples were kept on dry ice for the entirety of the experiment. Some 50 µL of chilled MeOH:H_2_O (1:1) was added to the microfuge tube containing the sample. Tissues were minced with small scissors in solution for two minutes, vortexed for 45 seconds, and centrifuged at 14,000 rpm for 20 min at 4 °C (Beckman Microfuge 18) to create a protein pellet. The supernatants were transferred to a newly labeled microfuge tube. In total, 50 µL of chilled acetonitrile–acetone–MeOH (8:1:1) was then added to the original microfuge tube, repeating the previous steps outlined above. The supernatants were transferred to the newly labeled microfuge tube and dried via a speed vacuum at room temperature. Two extraction blanks were prepared following the same steps as the biological samples.

#### 2.3.3. Untargeted Liquid Chromatography and Mass Spectrometry

Dried metabolite samples were reconstituted in 100 µL of HPLC-MS-grade water. Overall, 20 µL of internal standard (D-Glucose-1,2,3,4,5,6,6-d7) was added to each sample. The samples were sonicated for 25 min in an ultrasonic water bath for total solubilization, followed by centrifugation at 14,000 rpm for 5 min at 4 °C (Beckman Microfuge 18). 

Pooled quality controls (QCs) containing all compounds within the batch were run in separate HPLC-MS vials to account for reproducibility. Pooled QCs were created by combining 10 µL aliquots of each sample in new tubes.

Using a Thermo Scientific™ Vanquish™ Horizon Binary UHPLC, samples were subjected to fractionation and detection. An AccucoreTM VanquishTM C18+ Column (100 mm × 2.1 mm, 1.5 µm, Thermo Scientific™, Waltham, MA, USA) was used to separate compounds with a flow rate of 0.300 mL/min. Mobile Phase A consisted of acetonitrile with 0.1% formic acid (*v*/*v*). Mobile Phase B consisted of water with 0.1% formic acid (*v*/*v*). Column temperature was set to 40 °C and injection volume was set at 5 µL.

The samples were run using a Q Exactive™ mass spectrometer, coupled to a heated electrospray ionization (HESI) source. The spray voltage was set to 3.50 kV, capillary temperature to 256 °C, sheath gas to 48, aux gas to 11, sweep gas to 2, and S-Lens RF level to 50.0. The mass range was set to 67–1000 *m*/*z*; we set the resolution to 140,000 for full scans and to 35,000 for ddMS2. The AGC target was set to 3 × 10^6^ for full scans and 2 × 10^5^ for ddMS2s. The max injection time (IT) was 200 seconds for full-scan mode and 50 seconds for ddMS2. The number of microscans was 2, and normalized collision energy (NCE) was set to 20, 35, and 50. Samples were run in both positive and negative ion modes, separately. The parameters for negative mode were the same with the exceptions of the spray voltage, which was set to 2.50 kV, and the S-Lens RF level, which was set to 60.0.

#### 2.3.4. Metabolite Identification and Analysis

The raw scans were processed using Compound Discoverer 3.3. Extraction blanks were used to determine and correct reagent effects, allow for the creation of exclusions lists, mark background components, and filter the background components from the results table. Pooled QCs were used for compound identification. 

Metabolite peak area normalization was performed using the estimated protein amount created from the protein pellet during the extraction and analyzed through densitometry and ImageJ. Metabolites with duplicate peak areas were merged. The compounds identified in both positive and negative ionization mode were aligned. 

Metabolites and their respective normalized peak areas were uploaded to MetaboAnalyst 6.0. Data were transformed using log transformation (base 10) and scaled using the auto scale feature. Volcano plots were generated with a p value threshold of 0.05 and a fold-change threshold of 2.0. Significant compound IDs were entered into the metabolite ID conversion tool for pathway analysis. Metabolite KEGG identifications were processed using the KEGG Mapper Search tool with mmu as the organism code.

## 3. Results

**Figure 1:**
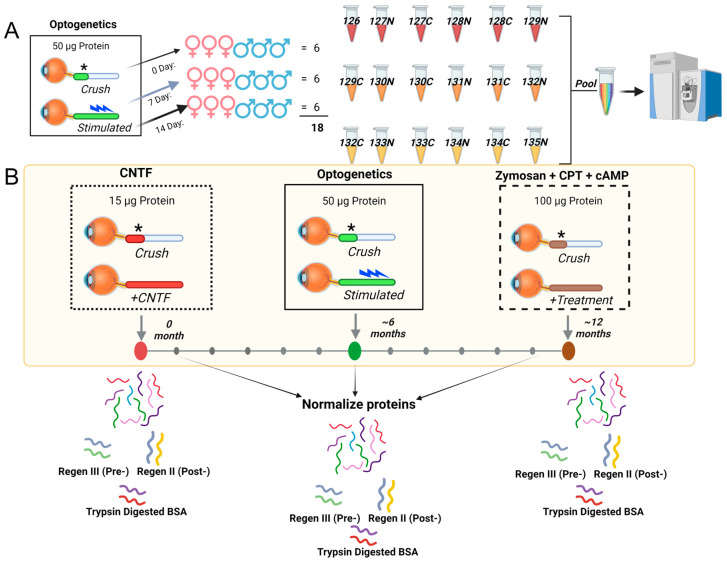
Schematic depiction of external peptide-driven multi-organism/multi-system protein normalization. (**A**) A schematic detailing inter-sample relative quantification, using commercially available TMTpro18plex (Thermo Scientific^TM^, Waltham, MA, USA) labeling of optic nerve tissue across three time points (0, 7 and 14 day) after optic nerve crush (crush site is denoted by *), with n = 6 animals at each point (equal male and female distribution). Rhythmic light stimulation post crushing to regenerate axons, indicated by a light blue symbol. (**B**) The normalization across experimental samples containing potentially varying protein amounts (15, 50, 100 µg total protein as examples) and sample arrival times (0, 6 and 12 months). Five unique peptides (Regen V) and trypsin-digested BSA were used for normalization. The Regen V consisted of Regen III peptides (SEB, LLO, CFB), added prior to extraction to measure extraction efficiency, and Regen II peptides (HH4B, A1315) were added post extraction as a composite measure of machine conditions, including ionization efficiency. Trypsin-digested BSA (100 pM) was spiked into each sample prior to mass spectrometry analysis for further normalization.

We present here a conceptual framework of proteome normalization (Figure 1) and the new term of “normativity” for metabolome and lipidome. Whereas robust relative quantification across samples can be achieved using labeled proteomics such as tandem mass tags (TMT), where protein abundances are determined by the summation of tagged unique and razor peptide group abundances [9] (Figure 1A), it is the normalization across multiple samples, each with multiple time points, where advances are needed (Figure 1B). With four time points (0, 7, 14, or 28 days after ONC), TMT enables relative quantification, with n = 6 samples distributed evenly among males and females in each of the three groups. We utilize 18 plex for these samples (Figure 1A). However, instances of multiple axon regeneration, promoted by different agents such as ciliary neurotrophic factor (CNTF) [10], rhythmic light stimulation in transgenic channel rhodopsin mice (optogenetics) [11], and zymosan and cyclic AMP analog CPT-cAMP [12], cannot be simultaneously compared with each other using relative labeled quantification, necessitating the peptide doping or spiking-in of peptides of known amounts in each sample (Figure 1B). We propose to normalize proteome in two tiers, that is, spiked-in unique peptides and a second additional tier with spiked-in trypsin-digested bovine serum albumin peptides. Spiked-in unique peptides for pre- and post-extraction normalization are referred to as Regen III and Regen II, respectively (Figure 1B, bottom part).

### 3.1. Proteomic Data Normalization by Regen Peptides

**Figure 2:**
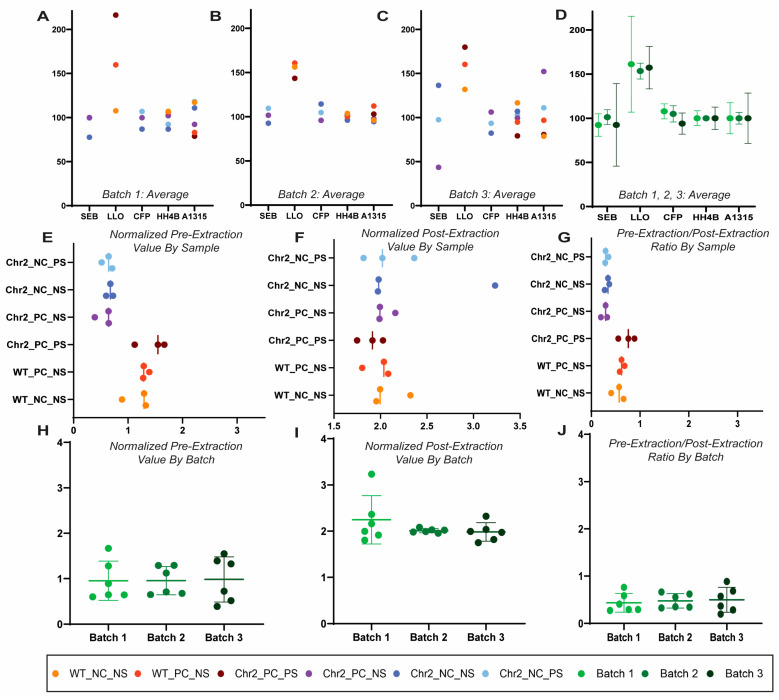
Normalization of protein abundance values. We used three independent batches of transgenic channel rhodopsin mice optic nerves stimulated by rhythmic light for axon regeneration (true biological replicates) to demonstrate normalization in three independent ensembles of protein samples, each with 18-plex relative quantification. Each batch of 18 optic nerve samples was spiked with a pre-determined amount of Regen V peptide for a final concentration of 36 µm Regen III (SEB, LLO, CFB) and 54 µm Regen II (HH4B, A1315). Scatter plots showing the regen peptide averaged abundances by biological replicates between all sample groups (see methods for details). Each sample group is denoted by color in batches 1 (**A**), 2 (**B**), and 3 (**C**). (**D**) A scatter plot showing the comparison of the average peptide abundances with standard deviation by biological replicate in each batch prior to normalization. Each batch is represented by a color as indicated. (**E**) The pre-extraction correction factor was calculated for each individual tag via multiplication of the peptide raw pre-extraction abundance value based on the spiked BSA concentration (µg), divided by the pre-extraction regen peptide BSA-normalized abundance value (µg), multiplied by the raw BSA abundance value. (**F**) The post-extraction correction factor was created for each individual tag through the multiplication of the post-extraction peptides raw abundance value by the spiked BSA concentration (µg), divided by the post-extraction regen peptides normalized abundance value (µg), which is multiplied by the raw BSA abundance value. (**G**) For each sample within each batch, the normalized value of the pre-extraction regen peptides was divided by the normalized value of the post-extraction regen peptides. The peptide abundances within each tag were multiplied by their individual correction factors for analysis. (**H**–**J**) Batch comparison (three independent batches of axon regenerating transgenic channel rhodopsin mice) of the individually normalized values for pre-extraction, post-extraction, and pre-extraction/post-extraction ratio. Each batch is represented by a color as indicated.

Raw and normalized pre-extraction and post-extraction peptide abundance values were compared to determine uniformity between the batches. The peptides were normalized individually on the basis of sample per batch to 100 pmol trypsin-digested BSA and subsequently with a fixed value of spiked-in Regen V peptides. There was variability in abundance values between batches, though they were extracted and analyzed at the same time (Figure 2A–D). Normalizing to a known value of spiked trypsin-digested BSA allowed for uniformity in the varying values between all three batches (Figure 2E–G). With the normalized values of the pre-extraction (Regen III) and post-extraction (Regen II) peptides, which together constitute Regen V peptides, a correction factor for each sample was calculated (Figure 2H–J). In an ideal scenario, the correction factor would be approximately 0.66, but here was calculated to be approximately 0.5, indicating a 75.7% extraction efficiency.

### 3.2. Normativity of Metabolome and Lipidome

**Figure 3:**
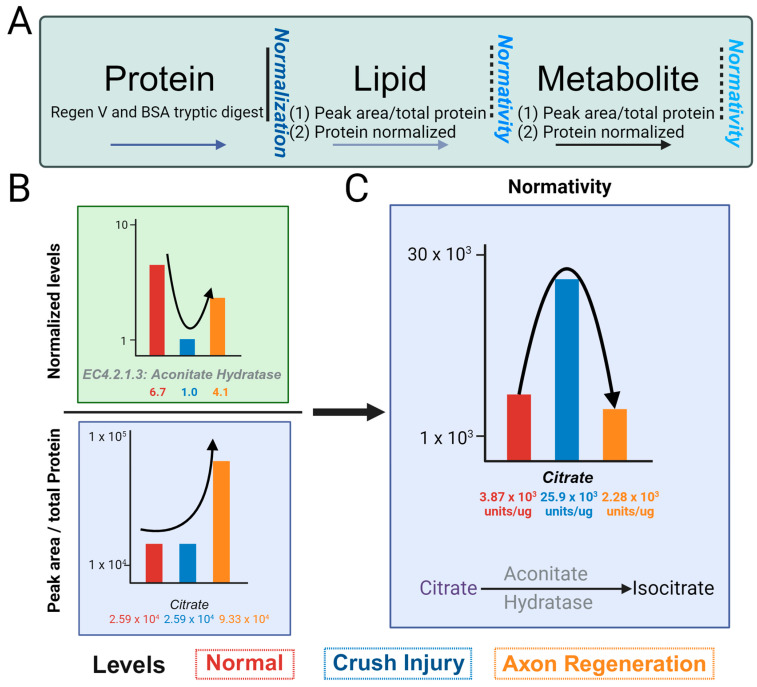
Concept of normativity (metabolites/lipids), utilizing protein normalized values. (**A**) Schematic depicting normalized protein values and normativity of metabolites and lipids. The relative number of metabolites and/or lipids per total protein content subsequently adjusted to normalized value of one protein value (or average of multiple pertinent protein values) is the normativity of metabolites and/or lipids. Selected proteins for normativity assessment should be pertinent to a feature of the samples, such as consistent amount across samples or housekeeping proteins (such as GAPDH or β-actin) or an initial decrease and subsequent increasing trend over a time course as would be applicable to optic nerve crush (ONC) and axon regeneration. (**B**) Depiction of normativity calculation for citrate. Citrate is experimentally a robustly identified metabolite. The metabolite value is divided by its corresponding normalized protein value of aconitate hydratase, showing a decrease in ONC, and the subsequent increase with axon regeneration, resulting in a normative correlation between the protein and metabolite levels of citrate. The levels of normal, ONC, and axon regeneration are represented using red, blue, and orange bars, respectively. (**C**) Illustration of the normative values of citrate determined to the normalized values of aconitate hydratase in normal, ONC, and axon regeneration.

Whereas RNA and proteins can be normalized with only a small subset of entities, this cannot be performed for small molecules due to non-linearity across, even within a small range of the spectrum. We will term the normativity (Figure 3A) of metabolome and lipidome as the number of small molecules (metabolome and lipidome) compared to the amount of specific proteins in the protein pathways. To assess normativity across metabolome and/or lipidome, we compared our normalized protein concentrations to the respective relative abundance values of the metabolome and lipidome within the glyoxylate and sphingolipid pathways. We normalized the aconitate hydratase using the Regen V approach (Figure 3B), as indicated. The values for aconitate hydratase, noted below as 6.1, 1.0 and 4.1 µg, are for the uncrushed control (channel rhodopsin mice), ONC, and case with regeneration promoted by optogenetic light stimulation. Thus, aconitate hydratase level undergo a decrease in ONC and is increased in regeneration. The citrate level without the application of normativity (peak area normalized by total protein in the sample) shows 2.59 × 10^3^, 2.59 × 10^3^, and 9.33 × 10^3^ relative peak areas units/µg of total protein in control, ONC, and regeneration samples, respectively, as indicated (Figure 3B, bottom panel). The normative level of citrate, determined using aconitate hydratase levels, shows a different trend than the normalized values of citrate (Figure 3C). 

**Figure 4:**
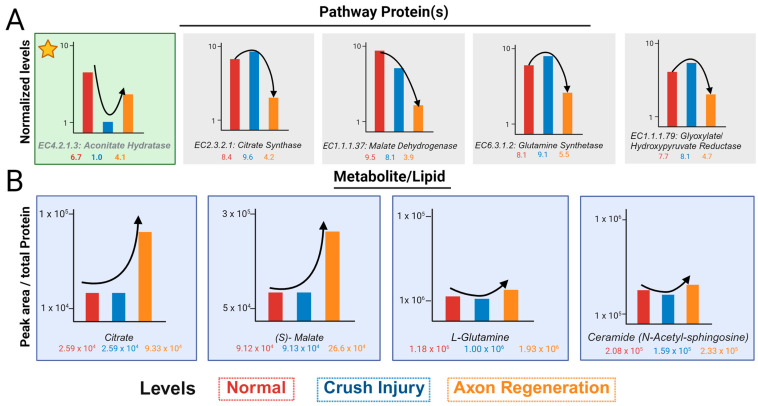
Schematics depict normalized levels of proteins and non-normative metabolite levels. (**A**) A schematic displaying the normalized protein values by BSA tryptic digest peptides and Regen V for each significant protein found within the glyoxylate metabolism. The protein denoted in green with the star, follows the ideal protein level trend before and after optic nerve crush injury. (**B**) A schematic illustrating the trend of the metabolite and lipid peak areas normalized by total protein of the sample before proteomic normativity was applied. Different treatments are represented as normal (red), optic nerve crush injury (blue), and axon regeneration (orange).

The normalized values of proteins in the glyoxylate pathway show one of two trends. The first is a decrease in ONC, followed by an increase in axon regeneration (Figure 4A). This is shown by aconitate hydratase (marked by asterisk) and is consistent with the loss of axons in the ONC and their growing back in regeneration. Most other proteins show an increase in ONC, followed by a decrease in regeneration (Figure 4A). In contrast, the peak area normalized by the total protein contents of most metabolites, with the exception of Ceramide-N-acetyl-sphingosine, shows a lack of appreciable change in ONC and an increased level in regeneration (Figure 4B). Ceramide-N-acetyl-sphingosine levels are similar to that of aconitate hydratase with respect to the trend and this is consistent with axon loss and gain during ONC and regeneration, respectively. 

### 3.3. Pathway Analysis

**Figure 5:**
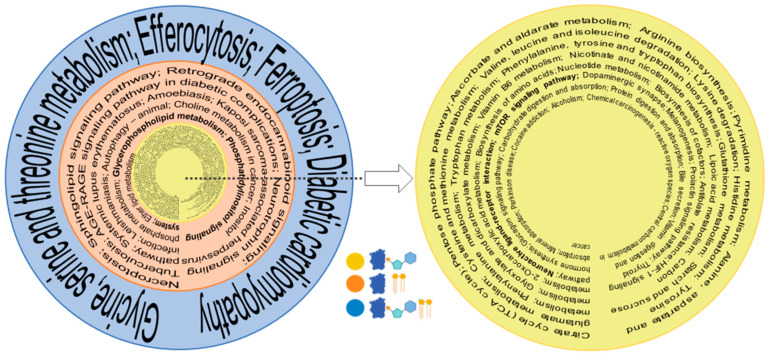
Pathways with combinatorial omes. Significant metabolic pathways were identified individually by KEGG for proteome, lipidome, and metabolome. From the largest to smallest omic correlations, the yellow circle represents commonly identified pathways, combining metabolome and the proteome (most numbers); the orange circle depicts that arrangement with the proteome and metabolome; and the blue circle represents the proteome, metabolome, and lipidome (least numbers). Previously known pathways relating to axon regeneration are bolded. The blue, yellow, and light blue symbols (bottom corner) depict protein, lipid, and metabolites.

Using KEGG mapper, we also investigated the pathways that correlated with the biological functions of identified entities in the proteome, metabolome, and lipidome. The consolidation of pathways based on the commonality between the omics (Figure 5, Supplemental Table S1) focuses on important trends. Whereas single ome pathways have a much longer list of identified pathways, the number of pathways shrink when more omes are combined; that is, the highest ome combinations have the lowest number of pathways and filter out select pathways for the investigation of their multi-nodal involvement in axon regeneration. The proteome and metabolome have the largest list of from among the two combined ome pathways, as would be expected. The combination of three pathways has the smallest number of pathways. Several pathways known for axon regeneration in prior studies are bolded (Figure 5), attesting that old pathways are identified but new pathways emerge from multi-omics comparison. There was a total of 44 common pathways between proteomics and metabolomics, 16 between proteomics and lipidomics, and 4 between proteomics, metabolomics, and lipidomics (Figure 5, Supplemental Table S1).

## 4. Discussion

Axons are the specialized appendages of the neurons that ensure neuron-to-neuron connectivity and fast electrical signal transduction across them. Axon loss is associated with the loss of motion control and other functionalities due to spinal cord injuries and the loss of vision in glaucoma, traumatic neuropathies, and trauma-induced secondary glaucoma. Glaucoma affects a sizeable patient population, currently estimated to be more than 80 million people worldwide [13]. The individuals suffering from such blindness as well as spinal cord injuries will benefit from axon regeneration therapies. Several decades of research altered the paradigm so that central nervous system (CNS) axon regeneration is considered possible [14,15,16], moving from the old axiom where that they are impossible to regrow [17,18,19]. The RGCs reside within the retina, while long axons traverse from the retina via the optic nerve to the brain. The RGCs survive for a long time, even after losing the axons, and provide opportunity to regrow with a singular clinical assessment, an objective visual function, or visual acuity, thus rendering them suitable for clinical trials. Currently, optimal axon regeneration is achieved by gene manipulation [15] and is best achieved in very young animals [20]. The repertoire of molecules that can be used for axon regeneration in adult animals with relevance for the human patient population needs to be expanded. Multiple-organism multi-omics comparisons, where regeneration is induced by various pharmacological means, are likely to aid in finding molecules that will help the current age-related critical barrier [20] in axon regeneration. 

Multi-omics comparisons necessitate the consideration of system-level standardization for the portability of molecular understanding from model to model and finally to humans. With respect to proteomes, labeled quantitative proteomics have greatly expanded our ability for relative quantification. Current 18-plex TMT enables the comparison of 18 simultaneous samples [9]. However, they are insufficient for multi-system comparison (Figure 1B). For axon regeneration studies, we devised the concept of Regen V peptide to enable pre- and post-protein extraction normalization, with an added second tier containing BSA tryptic digests to add further robustness. Two-tier bioinformatics of global labeled peptide analysis and local database searches for Regen V and BSA tryptic peptides may result in ease of abundance determination (Figure 2) across several samples. Using different batches of optogenetically induced axon regeneration for a channel rhodopsin transgenic mouse, we established the basic premise of this approach (Figure 2). 

The transcriptome as well as proteome are usually normalized based on a handful of RNA and proteins. When used with sufficient unique (and razor) peptides [9], a handful of housekeeping proteins such as GAPDH or β-actin help to normalize the protein data. For the regeneration of axons, a few markers such as RPBMS, Tuji, and GAP43 are bellwethers of regeneration [21,22,23,24,25]. With ONC, all axons are lost, albeit not immediately, but practically we see losses within a few hours, by which time the researchers are able to euthanize and dissect the animals [26,27]. Consistent with this trend, a lipid and a protein should show an immediate decrease in the ONC and a subsequent increase during regeneration. With an increased time point of regeneration, such levels should undergo a progressive increase. Our analysis shows that while aconitate hydratase is consistent with the expected trend, there is a decrease in concentration with crush injuries and an increase in regeneration when compared to the control (Figure 3 and Figure 4). 

A decrease in the activity of aconitate hydratase as observed in ONC would lead to a reduction in the utilization of citrate. In conjunction with the increase in citrate synthase, it is expected that the citrate level will correspondingly increase (Figure 3B,C). This did not occur because of the reduction in the oxaloacetate substrate at the malate checkpoint in the Krebs cycle, which resulted in an accumulation of malate (Figure 4B), making the system unable to maintain the citrate levels. Determining whether malate utilization, resulting in higher ATP formation in several regeneration systems, can lead to high citrate implementation will be a strong candidate for future axon regeneration and demonstrates the utility of standardized comparison. Glyoxylate pyruvate reductase was included to show consistency within its group—the enzyme increased during crushing and decreased in regeneration. The glutamine levels are related to glutamine toxicity but remain inconsistent across the group levels. It is known that glutamine is directly associated with neurodegeneration—in our findings, glutamine decreases with injury and increases during regeneration, despite glutamine synthase showing the opposite trend. These issues are too complex to account for in a singular investigation. In addition to biological complexity, a metabolite is utilized by several branch pathways. Precursors are contributed in various ways including several cell-related analytical problems such as ionization in the mass spectrometer. Further, proper capture of all ionized species in positive and negative ion modes could be contributory to anomalies and cannot be ruled out either. The standardized comparison brings forth a few issues for future axon regeneration studies, such as the need for the analysis of fractionated cells and organelles because tissue-level metabolites and proteins can be contributed by several cells, not just by axons and the determination of metabolomic flux. We present here an advanced approach for normalizing the protein levels and obtaining the normativity of lipid and metabolite levels. This can be utilized for several regenerative systems simultaneously, as well as for fractionated cells and organelles. To better understand normativity across systems, the candidate proteins, lipids, and metabolites need to be expanded and further analyzed to find molecules that may help to resolve the critical barrier of achieving axon regeneration across the age groups most applicable to currently suffering human beings [20]. One of the best regenerations is the phosphatase and tensin homolog (PTEN) knockout [16]. The evaluation of the robust PTEN knockout optic nerve regeneration model in multiple mouse strains has illustrated significant variability in regenerative response [28]. None of these studies take into account variability in optic nerve volumes among mouse strains and at different ages [29]. These findings further underscore that metabolomic/lipidomic normalization by total protein content is insufficient. Elucidating the differences among mouse strains, like human diversity, also necessitates improved protein and localized pathway normalization that can be applied to other omics.

A question arises as to which proteins should be utilized to achieve normativity. For most lipids and metabolites, it should be a protein that is uniquely present in axons such as GAP43, whose actual level is expected to go down in ONC and increases during regeneration. This also brings an important point about the fractionated cell analysis. Most lipids and metabolites are contributed by other cell types in the optic nerve, such as astroglial cells. Unless the actual data of lipids and metabolites are present from fractionated cells and axons, it will be impractical to utilize machine learning or artificial intelligence from current tissue omics datasets to arrive at correct conclusions. Regenerating axons are associated with some unique lipids, including ganglioside GM1 and GM3 [30], which undergo an increase and later a calibrated decrease in axon regeneration, with opposite trends in degeneration [31]. Ceramide N-Acetyl sphingosine is somewhat uniquely associated with axon membranes [30,32] and gangliosides, particularly, GM1 biosynthesis [33]. It is therefore expected that its levels will undergo a decrease in ONC and incremental subsequent increase in regeneration (Figure 4B), attesting the utility of standardized tissue-level multi-omics. Pathway analysis is an important aspect of this. Although lipid-metabolizing proteins hold significant importance, they constitute a comparatively smaller portion of the proteome. It is not surprising, then, that a combination of the proteome and metabolome provides a larger list than that of the proteome and lipidome (Figure 5). It is unsurprising that a number of previously known axon regeneration-promoting pathways are identified in optogenetically induced axon regeneration, as represented by bolded pathways in the combinations. However, all three ome comparisons help to identify new pathways that were not previously linked to optic nerve axon regeneration (Figure 5). Several pathways such as mTOR [15,34] were identified in combinatorial of proteomics and metabolomics analysis that had been previously linked to CNS, as well as peripheral axon regeneration (smallest innermost circle, Figure 5). Glycerophospholipid and shingolipid pathways [35,36] were also found in association with one another when combinations of proteomics and lipidomics were analyzed. It is no surprise that efferocytosis, identified with proteome, metabolome, lipidome combinatorial analysis, is associated with neuroinflammation resolution. They induce neurorepair (outermost circle, Figure 5) and are important for optic nerve axon regeneration [37,38], as demonstrated with zymosan-induced axon regeneration [39]. Similarly, the ferroptosis pathway identified in three ome combination analysis has been implicated in neuronal survival [40] and postinjury signal stabilization [41], but remains to be fully explored in axon regeneration. The combinatorial analysis for pathways thus suggests that, in addition to previously explored pathways, new pathways may be identified in such analyses. It will be interesting to analyze multi-organism pathways to assess whether they provide important clues enabling identification of a blueprint for axon regeneration across different organisms and eventually for humans CNS injuries.

## 5. Conclusions

The standardized comparison of data, including the normalization of values, is a critical aspects of various research fields, significantly influencing the interpretation and comparability of study outcomes. To eliminate technical variations and biases from the data, researchers must consider normalized data to derive meaningful conclusions and confirm the validity of their analyses [42,43]. A lack of or the use of incorrect normalization methods can lead to false conclusions and the misinterpretation of results; therefore, it is crucial to carefully choose an appropriate normalization strategy, tailored to the specific experimental design [44]. The utilization of normalized data allows for the comparison of study results against established benchmarks, magnifying the understanding and interpretation of the results [42]. Normative data play a crucial role in establishing an accurate depiction of timepoint multi-omics analysis and serves as a means to connect disparate organismal multi-omics datasets. By addressing the relationship between descriptive data and normative arguments, researchers can enhance the transparency and precision of their research practices [45,46]. By using this framework, a more accurate quantified proteomic profile can be created as the base for multi-omics comparisons. This requires the use of multiple standards within samples. Examples include trypsin-digested protein standards such as BSA and peptide standards (Regen V) for protein extraction and ionization standardization. Quantified metabolomic, transcriptomic, and lipidomic datasets should be compared using relevant pathway proteins. Further consideration should be applied to the rate of “ome” degradation/turnover and how many datasets and replicates would be necessary to establish a method that is closer to real profiling. Organismal differences in these rates should also be considered. Data normativity serves as a guiding principle for researchers, enabling them to draw valid conclusions, compare results effectively, and align their analyses with established norms and standards. By considering research, scholars can enhance the robustness and applicability of their findings across various disciplines. Proper data normalization [43] not only enhances the quality and reliability of research outcomes but also boosts the statistical power of analyses, enabling larger-scale experiments in various fields like integrative multi-omics [47]. Therefore, we must thoughtfully consider and validate normalization methods to derive valid and meaningful conclusions from multi-omics data.

## Data Availability

The proteomic mass spectrometry data has been deposited to the Proteome Xchange Consortium: http://proteomecentral.proteomexchange.org (accessed on 12 January 2024) via the MASSIVE repository with the dataset identifier PXD047136. The metabolomic and lipidomic mass spectrometry data has been deposited via metabolomics workbench with the dataset identifiers ST002111 and ST001381. We acknowledge using BioRender for constructing Figure 1, Figure 3 and Figure 4.

## Supplementary Materials

The following supporting information can be downloaded at: https://www.mdpi.com/article/10.3390/biom14070735/s1, Table S1: List of Common Pathways, Related to Figure 5.

## References

[1] Curcio M., Bradke F. (2018). Axon Regeneration in the Central Nervous System: Facing the Challenges from the Inside. Annu. Rev. Cell Dev. Biol..

[2] Alfadil E., Bradke F. (2023). Moving through the crowd. Where are we at understanding physiological axon growth?. Semin. Cell Dev. Biol..

[3] Pfeiffer R.L., Marc R.E., Jones B.W. (2020). Persistent remodeling and neurodegeneration in late-stage retinal degeneration. Prog. Retin. Eye Res..

[4] Pfeiffer R.L., Anderson J.R., Dahal J., Garcia J.C., Yang J.H., Sigulinsky C.L., Rapp K., Emrich D.P., Watt C.B., Johnstun H.A. (2020). A pathoconnectome of early neurodegeneration: Network changes in retinal degeneration. Exp. Eye Res..

[5] Masuda S., Tanaka S., Shiraki H., Sotomaru Y., Harada K., Hide I., Kiuchi Y., Sakai N. (2022). GPR3 expression in retinal ganglion cells contributes to neuron survival and accelerates axonal regeneration after optic nerve crush in mice. Neurobiol. Dis..

[6] Harvey B.M., Baxter M., Granato M. (2019). Optic nerve regeneration in larval zebrafish exhibits spontaneous capacity for retinotopic but not tectum specific axon targeting. PLoS ONE.

[7] Bernstein J.A., Khodursky A.B., Lin P.H., Lin-Chao S., Cohen S.N. (2002). Global analysis of mRNA decay and abundance in Escherichia coli at single-gene resolution using two-color fluorescent DNA microarrays. Proc. Natl. Acad. Sci. USA.

[8] Gygi S.P., Rochon Y., Franza B.R., Aebersold R. (1999). Correlation between protein and mRNA abundance in yeast. Mol. Cell. Biol..

[9] Weiner S., Sauer M., Visser P.J., Tijms B.M., Vorontsov E., Blennow K., Zetterberg H., Gobom J. (2022). Optimized sample preparation and data analysis for TMT proteomic analysis of cerebrospinal fluid applied to the identification of Alzheimer′s disease biomarkers. Clin. Proteom..

[10] Bei F., Lee H.H.C., Liu X., Gunner G., Jin H., Ma L., Wang C., Hou L., Hensch T.K., Frank E. (2016). Restoration of Visual Function by Enhancing Conduction in Regenerated Axons. Cell.

[11] Harvey F.C., Mendoza X., Liu Y., Lee R.K., Bhattacharya S.K. (2022). Labeled quantitative proteomics dataset of optogenetics induced axon regeneration in mice. Data Brief.

[12] Yin Y., Cui Q., Li Y., Irwin N., Fischer D., Harvey A.R., Benowitz L.I. (2003). Macrophage-derived factors stimulate optic nerve regeneration. J. Neurosci..

[13] Coleman A.L. (1999). Glaucoma. Lancet.

[14] Benowitz L.I., He Z., Goldberg J.L. (2017). Reaching the brain: Advances in optic nerve regeneration. Exp. Neurol..

[15] Park K.K., Liu K., Hu Y., Kanter J.L., He Z. (2010). PTEN/mTOR and axon regeneration. Exp. Neurol..

[16] Park K.K., Liu K., Hu Y., Smith P.D., Wang C., Cai B., Xu B., Connolly L., Kramvis I., Sahin M. (2008). Promoting axon regeneration in the adult CNS by modulation of the PTEN/mTOR pathway. Science.

[17] Arnemann J., Dieterich J.C. (1786). Ueber Die Reproduktion der Nerven.

[18] Arnemann J., Dieterich J.C. (1787). Versuche Über Die Regeneration an Lebenden Thieren/1 Über Die Regeneration der Nerven: Mit IV Kupfertafeln.

[19] Cruikshank W.E. (1797). Experiments on the Nerves, Particularly on Their Reproduction; and on the Spinal Marrow of Living Animals: From the Same Work. Med. Facts Obs..

[20] Geoffroy C.G., Hilton B.J., Tetzlaff W., Zheng B. (2016). Evidence for an Age-Dependent Decline in Axon Regeneration in the Adult Mammalian Central Nervous System. Cell Rep..

[21] Chintalapudi S.R., Djenderedjian L., Stiemke A.B., Steinle J.J., Jablonski M.M., Morales-Tirado V.M. (2016). Isolation and Molecular Profiling of Primary Mouse Retinal Ganglion Cells: Comparison of Phenotypes from Healthy and Glaucomatous Retinas. Front. Aging Neurosci..

[22] Kaneda M., Nagashima M., Mawatari K., Nunome T., Muramoto K., Sugitani K., Kato S. (2010). Growth-associated protein43 (GAP43) is a biochemical marker for the whole period of fish optic nerve regeneration. Adv. Exp. Med. Biol..

[23] Kwong J.M., Quan A., Kyung H., Piri N., Caprioli J. (2011). Quantitative analysis of retinal ganglion cell survival with Rbpms immunolabeling in animal models of optic neuropathies. Investig. Ophthalmol. Vis. Sci..

[24] Mesentier-Louro L.A., Zaverucha-do-Valle C., da Silva-Junior A.J., Nascimento-Dos-Santos G., Gubert F., de Figueiredo A.B., Torres A.L., Paredes B.D., Teixeira C., Tovar-Moll F. (2014). Distribution of mesenchymal stem cells and effects on neuronal survival and axon regeneration after optic nerve crush and cell therapy. PLoS ONE.

[25] Sharma T.P., Liu Y., Wordinger R.J., Pang I.H., Clark A.F. (2015). Neuritin 1 promotes retinal ganglion cell survival and axonal regeneration following optic nerve crush. Cell Death Dis..

[26] Daniel S., Clark A.F., McDowell C.M. (2018). Subtype-specific response of retinal ganglion cells to optic nerve crush. Cell Death Discov..

[27] Yang N., Young B.K., Wang P., Tian N. (2020). The Susceptibility of Retinal Ganglion Cells to Optic Nerve Injury is Type Specific. Cells.

[28] Wang J., Li Y., King R., Struebing F.L., Geisert E.E. (2018). Optic nerve regeneration in the mouse is a complex trait modulated by genetic background. Mol. Vis..

[29] Meehan S.D., Abdelrahman L., Arcuri J., Park K.K., Samarah M., Bhattacharya S.K. (2021). Proteomics and systems biology in optic nerve regeneration. Adv. Protein Chem. Struct. Biol..

[30] Chauhan M.Z., Arcuri J., Park K.K., Zafar M.K., Fatmi R., Hackam A.S., Yin Y., Benowitz L., Goldberg J.L., Samarah M. (2020). Multi-Omic Analyses of Growth Cones at Different Developmental Stages Provides Insight into Pathways in Adult Neuroregeneration. iScience.

[31] Chauhan M.Z., Valencia A.K., Piqueras M.C., Enriquez-Algeciras M., Bhattacharya S.K. (2019). Optic Nerve Lipidomics Reveal Impaired Glucosylsphingosine Lipids Pathway in Glaucoma. Investig. Ophthalmol. Vis. Sci..

[32] Pfenninger K.H. (2009). Plasma membrane expansion: A neuron′s Herculean task. Nat. Rev. Neurosci..

[33] Pitto M., Miglio A., Kirschner G., Leon A., Ghidoni R. (1991). Metabolism of semisynthetic single-chain GM1 derivatives in cerebellar granule cells in culture. Neurochem. Res..

[34] Huang Z., Wang W., Ma J., Li B., Chen J., Yang H. (2017). mTOR signaling pathway differently regulates central and peripheral axon regeneration. Acta Biochim. Biophys. Sin..

[35] Sbaschnig-Agler M., Ledeen R.W., Grafstein B., Alpert R.M. (1984). Ganglioside changes in the regenerating goldfish optic system: Comparison with glycoproteins and phospholipids. J. Neurosci. Res..

[36] Meehan S.D., Neag E., Bhattacharya S.K. (2023). Glycerophospholipid Analysis of Optic Nerve Regeneration Models Indicate Potential Membrane Order Changes Associated with the Lipidomic Shifts. J. Ocul. Pharmacol. Ther..

[37] Kalinski A.L., Yoon C., Huffman L.D., Duncker P.C., Kohen R., Passino R., Hafner H., Johnson C., Kawaguchi R., Carbajal K.S. (2020). Analysis of the immune response to sciatic nerve injury identifies efferocytosis as a key mechanism of nerve debridement. Elife.

[38] Estera L.A., Walsh S.P., Headen J.A., Williamson R.E., Kalinski A.L. (2023). Neuroinflammation: Breaking barriers and bridging gaps. Neurosci. Res..

[39] Feng Q., Wong K.A., Benowitz L.I. (2023). Full-length optic nerve regeneration in the absence of genetic manipulations. JCI Insight.

[40] Yin C., Ji Y., Ma N., Chen K., Zhang W., Bai D., Jia X., Xia S., Yin H. (2022). RNA-seq Analysis Reveals Potential Molecular Mechanisms of ZNF580/ZFP580 Promoting Neuronal Survival and Inhibiting Apoptosis after Hypoxic-ischemic Brain damage. Neuroscience.

[41] Xia M., Zhang Q., Zhang Y., Li R., Zhao T., Chen L., Liu Q., Zheng S., Li H., Qian Z. (2022). Growth Differentiation Factor 15 Regulates Oxidative Stress-Dependent Ferroptosis Post Spinal Cord Injury by Stabilizing the p62-Keap1-Nrf2 Signaling Pathway. Front. Aging Neurosci..

[42] Arraras J.I., Nolte S., Liegl G., Rose M., Manterola A., Illarramendi J.J., Zarandona U., Rico M., Teiejria L., Asin G. (2021). General Spanish population normative data analysis for the EORTC QLQ-C30 by sex, age, and health condition. Health Qual. Life Outcomes.

[43] Ejigu B.A., Valkenborg D., Baggerman G., Vanaerschot M., Witters E., Dujardin J.C., Burzykowski T., Berg M. (2013). Evaluation of normalization methods to pave the way towards large-scale LC-MS-based metabolomics profiling experiments. Omics.

[44] Robinson M.D., Oshlack A. (2010). A scaling normalization method for differential expression analysis of RNA-seq data. Genome Biol..

[45] Salloch S., Schildmann J., Vollmann J. (2012). Empirical research in medical ethics: How conceptual accounts on normative-empirical collaboration may improve research practice. BMC Med. Ethics.

[46] Salloch S., Wascher S., Vollmann J., Schildmann J. (2015). The normative background of empirical-ethical research: First steps towards a transparent and reasoned approach in the selection of an ethical theory. BMC Med. Ethics.

[47] Ewald J.D., Zhou G., Lu Y., Kolic J., Ellis C., Johnson J.D., Macdonald P.E., Xia J. (2024). Web-based multi-omics integration using the Analyst software suite. Nat. Protoc..

