# Supplementary material for: Concept of Normativity in Multi-Omics Analysis of Axon Regeneration
Source: Biomolecules. 2024 Jun 21;14(7):735. doi: 10.3390/biom14070735 (PMC11274927; doi:10.3390/biom14070735)
Supplement: Supplementary file 1 [file biomolecules-14-00735-s001.zip › biomolecules-3015664-supplementary.pdf]

Proteomics + Metabolites	Proteomics + Lipidomics	Proteomics + Lipidomics + Metabolomics
Citrate cycle (TCA cycle)	Necroptosis	Glycine, serine and threonine metabolism
Pentose phosphate pathway	**Sphingolipid signaling pathway**	Efferocytosis
Ascorbate and aldarate metabolism	Retrograde endocannabinoid signaling	Ferroptosis
Arginine biosynthesis	Tuberculosis	Diabetic cardiomyopathy
Pyrimidine metabolism	AGE-RAGE signaling pathway in diabetic complications	
Alanine, aspartate and glutamate metabolism	Neurotrophin signaling pathway	
Cysteine and methionine metabolism	Systemic lupus erythematosus	
Valine, leucine and isoleucine degradation	Amoebiasis	
Lysine degradation	Kaposi sarcoma-associated herpesvirus infection	
Histidine metabolism	Leishmaniasis	
Tyrosine metabolism	Autophagy - animal	
Phenylalanine metabolism	Choline metabolism in cancer	
Tryptophan metabolism	Inositol phosphate metabolism	
Phenylalanine, tyrosine and tryptophan biosynthesis	**Glycerophospholipid metabolism**	
Glutathione metabolism	**Phosphatidylinositol signaling system**	
Starch and sucrose metabolism	Ether lipid metabolism	
Glyoxylate and dicarboxylate metabolism		
Vitamin B6 metabolism		
Nicotinate and nicotinamide metabolism		
Lipoic acid metabolism		
Carbon metabolism		
2-Oxocarboxylic acid metabolism		
Biosynthesis of amino acids		
Nucleotide metabolism		
Biosynthesis of cofactors		
Antifolate resistance		
HIF-1 signaling pathway		
**Neuroactive ligand-receptor interaction**		
**mTOR signaling pathway**		
Dopaminergic synapse		
Melanogenesis		
Prolactin signaling pathway		
Thyroid hormone synthesis		
Glucagon signaling pathway		
Carbohydrate digestion and absorption		
Protein digestion and absorption		
Bile secretion		
Vitamin digestion and absorption		
Mineral absorption		
Parkinson disease		
Cocaine addiction		
Alcoholism		
Chemical carcinogenesis - reactive oxygen species		
Central carbon metabolism in cancer		
